# Encapsulation and dispersion of *Lactobacillus acidophilus* in a chocolate coating as a strategy for maintaining cell viability in cereal bars

**DOI:** 10.1038/s41598-021-00077-0

**Published:** 2021-10-15

**Authors:** Everton Luiz Lasta, Eduardo da Silva Pereira Ronning, Robert F. H. Dekker, Mário Antônio Alves da Cunha

**Affiliations:** 1 Programa de Pós-Graduação em Tecnologia de Processos Químicos e Bioquímicos, Universidade Tecnológica Federal do Paraná, Via do Conhecimento Km 01, Pato Branco, Paraná CEP 85503-390 Brazil; 2 Departamento de Química, Universidade Tecnológica Federal do Paraná, Via do Conhecimento Km 01, Pato Branco, Paraná CEP 85503-390 Brazil; 3 Grupo de Pesquisa em Tecnologia de Bioprocessos e Alimentos (GTBio), Universidade Tecnológica Federal do Paraná, Via do Conhecimento Km 01, Pato Branco, Paraná CEP 85503-390 Brazil; 4 Beta-Glucan Produtos Farmoquímicos EIRELI, Lote 24A, Bloco Zircônia, Universidade Tecnológica Federal do Paraná, Câmpus Londrina, Avenida João Miguel Caram 731, Londrina, Paraná CEP 86036-700 Brazil

**Keywords:** Biotechnology, Microbiology

## Abstract

Flour from *Pereskia aculeata* leaf and green banana were used as ingredients in the formulation of a cereal bar with added *Lactobacillus acidophilus* LA02-ID-1688. Encapsulation in a calcium-alginate hydrogel reinforced with magnesium hydroxide was used as a strategy to protect the probiotic cells under gastrointestinal conditions and to prolong shelf-life. The results are relevant especially for maintaining cell viability during shelf-life; a challenge for the food industry in relation to dry probiotic products. Encapsulation promoted the protection of probiotic cells in simulated gastric and intestinal conditions, allowing the maintenance of high viable cell counts (> 10 log CFU, colony forming unit). Encapsulation also contributed to cellular protection under extreme temperature conditions, with reductions of cell viability of < 1 logarithmic cycle when the capsules were subjected to 55ºC/10 min. Even at 75ºC/10 min, encapsulation protected the probiotic cells 3-times greater than the free-cells. The food bar proved to be rich in dietary fiber (19 g 100 g^−1^), lipids (12.63 g 100 g^−1^) and showed an appreciable protein content (5.44 g 100 g^−1^). A high viable probiotic cell count on storage over 120 days (12.54 log CFU) was observed, maintaining a probiotic survival rate > 90% and viability levels sufficient to promote health benefits.

## Introduction

Cereal bar snacks are well known for their practical consumption, ease of transport and the possibility of a fast energy supply, which makes them attractive to consumers. The global cereal bar market is expected to reach $16.9 billion by 2025, with a compound annual growth rate of 6.17% in the period between 2019 and 2025^1^, and there is a growing demand in this sector for physiological functional food products, rich in fiber, protein and probiotics bearing potential health benefits.

In the formulation of cereal bars, different ingredients with nutraceutical potential have been used, which enrich the nutritional quality of the product and promote health benefits to the body. Cereals such as oats contribute to the proper functioning of the gastrointestinal system through their content of dietary fibers that includes the β-glucans^2^. Green banana flour, another ingredient, is rich in resistant starch^3^ and phenolic compounds; the first being converted by beneficial gut microbiota into butyrate, a short-chain fatty acid source of energy^4^. *Pereskia aculeata* Miller leaf flour is rich in protein that contains all of the essential amino acids, and is rich in fibers, minerals (phosphorus, calcium and iron) and vitamins C, A and B complex^5^.

*P. aculeata* (Barbados gooseberry plant) is a non-conventional edible plant, native to South America that belongs to the Cactaceae family and Pereskioideae subfamily^6^. In Brazil it is popularly known as *ora-pro-nobis* (from the Latin “*pray for us*”) and its leaves are used in the cuisine of some regions, since they have considerable nutritional value^7^. Often the leaves of the plant have been called “*poor-man’s meat*”, as it can be the main source of protein available to low-income communities in some poor regions of the country. Its leaves can be used for the nutritional enrichment of different food products^6^, and in this sense, the development of food products containing *P. aculeata* leaf flour could stimulate the commercial cultivation of the plant and create a new agricultural market niche.

A major challenge for the food industry is to maintain the viability of probiotic bacteria in low moisture and low water activity products during shelf-life. Another limitation in functional foods containing probiotics is the loss of cell viability during gastrointestinal transit^8^, which can prevent adequate amounts (10^9^ CFU/per daily portion) of probiotics reaching the intestine by the end of the product’s shelf-life according to the Codex Alimentarius Commission^9^.

Cell encapsulation is a well-established strategy to protect probiotics against adverse technological and environmental conditions^10^. In the encapsulation of microbial cells, techniques based on procedures using physical (extrusion, freeze drying, fluidized bed coating, spray chilling, spray drying, supercritical precipitation and solvent evaporation), physicochemical (coacervation, liposomes and ionic geothermal) and chemical (interfacial polymerization and complexation by molecular inclusion) methods have been used^11^. Cellular encapsulation in alginate-based hydrogels is commonly used to protect microorganisms against adverse environmental conditions, due to alginate gelling rapidly and contributing to preserve the viability of the entrapped microorganisms^12^. Calcium alginate spheres are simply obtained by gelling a solution of sodium alginate with a divalent salt (commonly, calcium chloride), which crosslinks the alginate macromolecule to form calcium alginate^13,14^. The incorporation of magnesium hydroxide in appropriate concentrations in the hydrogel spheres contributes to greater cell protection under low pH conditions, such as occurs in the gastric environment^13^.

The aim of this study was to encapsulate the probiotic bacterium *Lactobacillus acidophilus* strain LA02 ID 1688 in a calcium alginate hydrogel containing magnesium hydroxide, as a strategy to protect against adverse conditions of the gastrointestinal tract and heat, and during storage. Additionally, the encapsulated probiotic was added as a chocolate topping to a cereal bar enriched with flour from *P. aculeate* leaf and green banana, with the objective of obtaining a product with attractive nutritional and functional potential. The study included the nutritional characterization of the ora-pro-nobis flour and its profile of amino acids and lipids; the immobilization of the probiotic in a calcium alginate hydrogel; the evaluation of the protective capacity of the hydrogel on the probiotic *L. acidophilus* LA02 ID 1688 under simulated gastric and intestinal conditions; and the development of the cereal bar and the characterization of its probiotic potential.

## Methods

### Ingredients and microorganisms used in the manufacture of the cereal bar

The ingredients used in the formulation of the cereal bar were purchased from a local market. *Pereskia aculeata* flour was supplied by the company Proteios Nutrição Funcional Ltda (Ribeirão Branco, São Paulo, Brazil). The lyophilized commercial strain of *Lactobacillus acidophilus* LA02 ID 1688 was from Probiotical S.p.A. (Novara, Piemonte, Italy) and kindly provided by the company COANA Importação e Exportação Ltda (Florianópolis, Santa Catarina, Brazil).

### Preparation of probiotic inoculum

The probiotic strain was activated by submerged cultivation. Three successive cultivations were carried out in MRS (Man, Rogosa and Sharpe) Broth (Sigma-Aldrich, St. Louis, MO, USA) in a TE4200 shaker incubator (Tecnal, Piracicaba, Brazil) at 37 ºC (150 rpm) for 15 h. The culture medium was prepared following the manufacturer's instructions and sterilized in an autoclave at 121 °C for 15 min. The bacterial isolate was activated (first cultivation) in MRS medium by transferring 1 g of the lyophilized culture to 100 mL of MRS broth in a 250-mL Erlenmeyer flask and cultivated as described above. The bacterial cells were then recovered aseptically by centrifugation (1592×*g*, 20 min), and resuspended in 40 mL of 1% (w/v) peptone-water. A volume of 10 mL of the microbial suspension was inoculated into 90 mL of MRS broth, and again cultured (second cultivation) in a shaker incubator. The third cultivation occurred under the same conditions as per the second activation stage, with the cells recovered aseptically by centrifugation (1592×*g*, 20 min). The recovered cells were washed twice with 40 mL of peptone water and resuspended in peptone water to obtain a cell suspension with a minimum concentration in the order of 16 log CFU mL^−1^. The numbers of bacteria in the cell suspension was determined by counting on plates in MRS Agar medium (37 °C, 72 h), using the *Pour Plate* inoculation technique^13^.

### Encapsulation of the probiotic agent

The probiotic strain was entrapped in calcium alginate spheres (micro-beads). A solution of 2% (w/v) sodium alginate in phosphate buffer (5 mmol L^−1^, pH 7.0) was prepared and kept under refrigeration at 5 °C for 24 h for the complete hydration of the biopolymer. Magnesium hydroxide (0.1%, w/v) was added to the sodium alginate solution, and the mixture stirred for 15 min. The mixture was sterilized (121 °C, 15 min), allowed to cool to room temperature, followed thereafter by adding the probiotic cell suspension, in order to obtain a probiotic cell count of at least 12 log CFU. The hydrogel beads were obtained by allowing a suspension of sodium alginate—probiotic cells to drop into a sterile solution of calcium chloride (0.68 mol L^−1^) under agitation, according to Arenales-Sierra et al.^13^ with adaptations. The system was assembled inside a laminar flow cabinet to promote a contamination-free environment during bead formation. A TE-PB-01-mini peristaltic pump (Tecnal, Piracicaba-SP, Brazil), sterile colorless silicone tubing and a disposable syringe needle (0.80 mm × 30 mm, 21G) was used to produce the hydrogel spheres. The flow rate of the pump was 5 mL min^−1^, and the spheres obtained were kept in the CaCl_2_ (0.68 mol L^−1^) solution for 30 min at 5 ºC for gelation of the hydrogel. Under aseptic conditions, the cured spheres were washed 3 times with sterile distilled water, drained in a 24 cm diam. stainless steel sieve, frozen at − 18 ºC, and lyophilized in a L108 freeze-drier (Liobras, São Carlos, São Paulo, Brazil).

### Ingredients and formulation of the cereal bar

The cereal bar was formulated by mixing a blend of dry ingredients (rice flakes, oat flakes, pecan nuts, green banana flour, *P. aculeate* leaf flour, Brazil nuts, banana-raisins, raisins, brown sugar and potassium sorbate as preservative) with a binding syrup in a ratio of 65:35 (w/w). The agglutination binding syrup was concentrated by boiling to 80 ºBrix, and the blend of dry ingredients was mixed in the syrup to 55 ºC. The mixture was then placed on a stainless-steel tabletop for laminating.

Vegan chocolate was melted in a water bath at 65 °C, then cooled to 45 °C, and used to prepare the topping to coat the cereal bar. A proportion of calcium alginate spheres (1%, w/w) containing the encapsulated probiotic cells was added to the chocolate topping to obtain a cell count of ~ 13 log CFU g^−1^. After cooling, the laminated dough was cut into ~ 20 g portions, which were in turn covered with the chocolate topping (72%, w/v cocoa) containing the encapsulated probiotic. The cereal bars produced were packed in sealed aluminum foil and plastic film to be stored until characterization, and for cell viability analysis over the shelf-life.

### Evaluation of the cell encapsulation efficiency

A one-gram portion of gel spheres was dissolved in 10 mL of 0.2 mol L^−1^ sodium citrate solution (pH 6.0) while vortexing^14^. Serial dilutions were performed and inoculated by the *Pour Plate* method (1 mL) on plates containing MRS-agar medium. The plates were inverted and incubated at 37 ºC for 72 h. Encapsulation efficiency was considered to be the combination of the probiotic's trapping effectiveness and its survival during the extrusion procedure, and was determined from Eq. (1) ^15^.

1 $$EE \left(\%\right)=\frac{\mathit{Log}N}{Log{ N}_{0}}\times 100$$

where *EE* (%) is the efficiency of the encapsulation; Log N is the number of logarithmic cycles of viable cells trapped in the gel spheres, and Log N_0_ is the number of logarithmic cycles of free cells present in the cell suspension.

### Morphological evaluation of the encapsulated probiotic

Micrographs were obtained by scanning electron microscopy (SEM) and optical microscopy (USB digital microscope c1335, KKmoon, China). The samples were placed on carbon tape and the SEM images were obtained on a bench electron microscope model TM3000 (Hitachi, Irving, TX USA) at magnifications of 100×, 600× and 1200×, using voltages from 5 to 15 kv. For the evaluation of the diameter of the encapsulated spheres, the spheres were placed on surfaces that were smooth and matted, and measurements taken after calibration according to the manufacturer's recommendations. Fifty spheres were analyzed and the diameters recorded on the USB digital optical microscope. Images of the encapsulated surface were obtained at magnifications of 10× and 100×.

### Viability assessment of free and encapsulated probiotic cells under simulated gastric and intestinal conditions, and upon heat treatment

Simulated gastric juice (SGJ) was prepared with a mixture comprising 0.9 g 100 mL^−1^ sodium chloride, 0.3 g 100 mL^−1^ pepsin and the pH adjusted to 2.0 with hydrochloric acid (1 mol L^−1^). Probiotic capsules (1 g) were mixed in 10 mL of SGJ, and the mixture incubated in a shaker (50 rpm) at 37 °C for 5, 30, 60 and 120 min^16^. After incubation, the spheres were collected on a stainless steel sieve, solubilized in 10 mL of 0.2 mol L^−1^ sodium citrate solution (pH 6.0), and cell viability was evaluated by the *Pour Plate* inoculation technique and counting cells grown on MRS-Agar plates at 37 °C for 48–72 h. A similar assay was conducted using free cells to compare the viability of free and encapsulated probiotic cells under similar conditions. A volume of 1 mL of cell suspension with a viable cell count of 9.26 log CFU mL^−1^ (adjusted using the McFarland scale) was dispersed in 10 mL of SGJ solution. All tests were conducted in triplicates.

In the preparation of the simulated intestinal juice (SIJ), 0.65 g 100 mL^−1^ NaCl, 0.0835 g 100 mL^−1^ KCl, 0.022 g 100 mL^−1^ CaCl_2_, 0.1386 g 100 mL^−1^ NaHCO_3_ were mixed and the pH adjusted to 7.5 with 1 mol L^−1^ sodium hydroxide solution. The SIJ solution was diluted to a final concentration of 0.3% (v/v) with water, and tests was carried out in triplicate. A 1 g portion of encapsulated probiotic cells was mixed with 10 mL of SIJ solution and the mixture incubated under gentle shaking (50 rpm) at 37 °C for 60 and 120 min^16^. After incubation and solubilization of the spheres, cell viability was assessed as described above. A similar assay with free cells was conducted using 1 mL of cell suspension (9.26 log CFU mL^−1^) dispersed in 10 ml of SIJ.

In the heat treatment experiments, free and encapsulated probiotic cells were kept at the following temperatures 55 ºC, 65 ºC and 75 ºC for 1 and 10 min^16^. One gram of encapsulated cells or 1 mL of a free cell suspension (9.26 log CFU mL^−1^) was dispersed in sterile distilled water and maintained at the pre-established temperatures and times. The viability of free and encapsulated cells was determined and compared.

### Fourier transform—infrared spectroscopy (FT-IR) analysis

Attenuated total reflection Fourier transform-infrared (ATR FT-IR) spectroscopy was employed for characterization of the capsules containing the probiotic cells. The spectra were obtained with a Frontier spectrophotometer (Perkin Elmer, Waltham, MA, USA) in the spectral region of 4000–400 cm^−1^, with a resolution of 4 cm^−1^ and 36 accumulations for each spectrum.

### Characterization of *Pereskia aculeata* leaf flour and the formulated cereal bar

The nutritional quality of the samples was assessed by analysis of proximal composition. Analyses were determined in accordance with the Official Methods of Analysis^17^, and included moisture and volatile contents (dried at 105 ºC, method 934.01); lipids (Soxhlet extraction, method 945.38); total protein (Kjeldahl, method 979.09); mineral residue (incineration at 550 ºC, method 923.03); and dietary fiber (enzymatic gravimetric method 985.29). Total carbohydrate content was determined as the difference between 100 and the sum of water, protein, total lipid, and mineral residue content. The energy value was estimated according to the Atwater conversion factors (proteins: 4 kcal g^−1^, carbohydrates: 4 kcal g^−1^ and lipids: 9 kcal g^−1^)^18^.

The amino acid profile of *P. aculeata* leaf flour protein was evaluated by High Performance Liquid Chromatography (HPLC) as described by White et al. (1986)^19^. The composition of fatty acids in the flour was determined by Gas Chromatography (GC) following the official method of analysis 996.06 of the Association of Analytical Chemists^17^.

The cell viability of the probiotic cells incorporated in the cereal bars was evaluated immediately after production, and thereafter, every 30 days during the storage period of up to 120 days. Under aseptic conditions, samples of cereal bars were suspended in sterile 1% peptone water (1:10 dilution) and ground in a mixer. Serial dilutions were prepared and aliquots of 1 mL were inoculated by the *Pour Plate* method using MRS-Agar medium. The plates were incubated at 37 ºC for 48–72 h^13^.

### Instrumental texture profile analysis

Instrumental texture was evaluated using a TA—XT2I texturometer (Stable Micro Systems Ltd, Godalming, Surrey, UK)^20^. For the analysis of the parameters: hardness, adhesiveness, cohesiveness, elasticity, gumminess and chewability, a cylindrical probe P/35 was used and the following conditions: pre-test speed of 1 mm s^−1^ and post-test of 1 mm s^−1^, compression speed of 2 mm s^−1^, penetration distance of 10 mm, time of 20 s and force of 0.1 N. In the analysis of shear, a probe Warner–Bratzler blade (HDP/BSW) and the following test conditions were used: pre-test speed of 1 mm s^−1^ and post-test of 1 mm s^−1^, test speed of 2 mm s^−1^, penetration distance of 30 mm, time of 80 s, and a force of 0.1 N applied. The tests were performed in duplicate using two compression cycles (force versus distance) to simulate chewing action of the product, and the cereal bar samples for testing were prepared in portions with dimensions of 3 × 3 × 1 cm (length × width × height).

### Statistical analysis

All analyzes were conducted in triplicate and results expressed as mean ± standard deviation. Means were compared by ANOVA and Tukey’s test (*P* < 0.05) using the GraphPad Prism^®^ 8.0 program, after checking the normal distribution of the results and homoscedasticity of the variance.

## Results and discussion

### Probiotic encapsulation efficiency

Preliminary tests evaluating the concentrations of sodium alginate (0.5%, 1%, 2% and 3%, w/v) in the preparation of the hydrogel showed that 2% (w/v) of sodium alginate resulted in uniform and resistant spheres, and allowed adequate flow in the apparatus used for extrusion (data not shown). In order to obtain hydrogel spheres of high cell density, a suspension of 15.5 log CFU mL^−1^ *L. acidophilus* LA02 ID 1688 was added to the alginate solution in the encapsulation process. This strategy was used to circumvent possible reduction in cell viability during the crosslinking step of the alginate in calcium chloride solution. Actually, this approach resulted in a 1.63 log CFU decrease in the number of viable cells in the hydrogel spheres compared to the probiotic cells added to the cell suspension prior to alginate bead formation. The reduction is associated with the detachment and non-entrapment of cells during the extrusion process, as well as the loss of viability during the lyophilization process of the spheres. It is important to mention that during the immobilization process, high concentrations of calcium chloride (0.68 mol L^−1^) were used for the crosslinking of sodium alginate, which may have contributed to cell injuries due to conditions of high osmolality. The freezing of the spheres prior to lyophilization may also have contributed to cell death.

Although cell death occurred, a high number of viable bacteria survived when entrapped in the hydrogel matrix (13.87 log CFU), which corresponded to an average encapsulation efficiency (EE) of 89.4%. Close values (82.80%) of efficiency were reported by Arenales-Sierra et al.^13^ when encapsulating *Lactobacillus casei* in calcium alginate spheres containing magnesium hydroxide, and following cell entrapment conditions similar to the present study. On the otherhand, lower values of EE were reported by Rather et al.^16^ when encapsulating *Lactobacillus plantarum* (EE: 72.48%) and *Lactobacillus casei* (EE: 62.54%) using the technique of double-layered spheres of calcium alginate.

It is important to note that different encapsulation techniques can promote different values for cell efficiency and viability. The cell viability of entrapped cells in hydrogels is associated with factors, such as osmotic stress conditions in the hydrogel crosslinking stage; the possibility of plasmolysis due to the formation of ice crystals during the freezing step prior to lyophilization; changes in the physical state of the membrane lipids and structures of proteins sensitive to lyophilization; changes in protein conformation during water removal; and cellular adaptability to the lyophilization process^21^. Other factors that can affect the probiotic encapsulation yield include the dimensions of the capsule, the nature of the wall material used, the microbial cell load, and the curing time in calcium chloride solution^22^.

### Resistance of free and encapsulated probiotic cells to simulated gastric and intestinal conditions, and to heat treatment

Probiotics having beneficial health effects must reach the region of the large intestine (colon) where they will colonize, resisting the adverse conditions of acidic pH of the stomach and the bile salts of the upper portion of the intestine. The survival of the probiotic bacteria (Table 1) as free cells after exposure to simulated gastric juice (SGJ) for between 5 and 120 min, ranged from 92.55 to 47.4% (decrease of 4.87 log CFU), while the survival rate for the encapsulated probiotic cells ranged from 96.76 to 88.68% (decrease of 1.57 log CFU).

**Table 1:** Viability of free and encapsulated probiotic cells under simulated gastric and intestinal conditions, and on heat treatment.

	Gastric simulation condition				Intestinal simulation condition			
Time (min)	Free cells (log CFU mL^−1^)	%^#^	Encapsulated cells (log CFU g^−1^)	%^#^	Free cells (log CFU mL^−1^)	%^#^	Encapsulated cells (log CFU g^−1^)	%^#^
0	9.26 ± 0.00^Ba^	0	13.87 ± 0.00^Aa^	0	9.26 ± 0.00^Ba^	0	13.87 ± 0.00^Aa^	0
5	8.57 ± 0.14^Bb^	7.45	13.42 ± 0.05^Ab^	3.24	–	–	–	–
30	7.38 ± 0.04^Bc^	20.30	13.36 ± 0.04^Ab^	3.68	–	–	–	–
60	5.46 ± 0.09^Bd^	41.04	13.23 ± 0.07^Ac^	4.61	6.39 ± 0.06^Bb^	30.99	12.41 ± 0.05^Ab^	1.05
120	4.39 ± 0.04^Be^	52.59	12.30 ± 0.04^Ad^	11.32	4.38 ± 0.03^Bc^	52.70	11.38 ± 0.04^Ac^	17.95

Heat treatment condition								
Time (min)	Temperature (^o^C)	Free cells (log CFU mL^−1^)*	%^#^	Encapsulated cells (log CFU g^−1^)*	%^#^			
0	25	9.26 ± 0.00^Ba^	0	13.87 ± 0.00^Aa^	0			
1	55	8.63 ± 0.05^Bb^	6.80	13.55 ± 0.03^Ab^	2.31			
1	65	7.38 ± 0.06^Bd^	20.30	12.71 ± 0.03^Ad^	8.36			
1	75	5.50 ± 0.04^Be^	40.60	11.38 ± 0.06^Ae^	17.95			
10	55	7.85 ± 0.02^Bc^	15.23	13.13 ± 0.05^Ac^	5.34			
10	65	5.59 ± 0.03^Be^	39.63	9.95 ± 0.01^Af^	28.26			
10	75	1.10 ± 0.07^Bf^	88.12	4.52 ± 0.06^Ag^	67.41			

^%#^Percent reduction in cell viability. Means followed by different capital letters differ statistically on the line; means followed by distinct lowercase letters differ statistically in the column (*p* < 0.05).

*Results of triplicate averages.

The cell encapsulation process contributed to the protection of the probiotic bacteria against the acidic conditions of the simulated gastric juice, maintaining close to 90% of viable cells over the 120 min of testing. It is interesting to note that, after one hour of exposure to SGJ, there was a reduction of only 4.6% in the viability of the encapsulated cells, in contrast to a 41% reduction in the viability of the free cells. Similar cell viability observations were reported by Arenales-Sierra et al.^13^ in gastric simulation tests of *L. casei* as free cells, and cells encapsulated in calcium alginate gel containing added magnesium hydroxide. They emphasized that the presence of magnesium hydroxide in the preparation of calcium alginate spheres contributed to the maintenance of a neutral pH inside the spheres, counteracting the acidic pH of the stomach, and hence increasing the viability of the probiotic cells.

Gu et al.^23^, in a study on the encapsulation of *Bifidobacterium pseudocatenulatum* G7, reported that free cells became unviable after 2 h under simulated gastric conditions. In the same study, when magnesium hydroxide was added to sodium alginate, this resulted in enhanced cell protection and promoted a low reduction in cell viability. Similarly, they reported that the free cells showed less resistance to simulated intestinal juice. On exposure to SIJ for 120 min there was a marked reduction in cell viability of around 53% (from 9.26 to 4.38 log CFU). On the other hand, the encapsulated cells showed greater resistance (82.05% viability) with a 17.95% reduction in cell viability (from 13.87 to 11.38 CFU) after 120 min.

The survival rate (47.3%) of the free probiotic cells observed in our study after subjection to SIJ for 120 min was considered low (Table 1). To obtain cell counts considered of probiotic value in a food product containing *Lactobacillus* (10^9^ CFU, in Brazil), it would be necessary to use very high counts of free probiotic cells (> 10^18^ CFU g^−1^) in the formulation of a cereal bar, considering the survival rate of the free bacteria. The data obtained are in agreement with those observed by Rather et al., (2017), who reported a very low survival rate of free cells of *Lactobacillus plantarum* NCDC201 when incubated in simulated intestinal juice, which was attributable to the low resistance of the cells to bile salts. The authors emphasized that microencapsulation of the probiotic cells contributed significantly to improve their survival on simulation to gastrointestinal conditions. Considering the reduction of cell viability in the free-form of probiotic cells along with gastric and intestinal simulation, a total reduction in the viable cell count of 7.16 log CFU was verified. This value corresponds to a reduction of 77% in cell viability, which would confer a load of viable cells with a cell count of approximately 2 × 10^2^ CFU. The above observations would indicate that *L. acidophilus* LA02 ID 1688 cells in the free-form would not reach the intestine in adequate probiotic quantities. On the otherhand, after gastric and intestinal simulations the encapsulated cells maintained a viability of 72.8% (1.26 10^10^ CFU).

The thermal resistance of probiotic cells is an important quality parameter, as several technological food-processing stages involve the application of heating. In the present study, the encapsulated probiotic cells showed high resistance to heat (Table 1). After a 10-min exposure to heating, the encapsulated cells showed viabilities above 94% at 55 ºC, and 70% at 65 ºC. The free probiotic cells, by contrast, proved to be more sensitive to heat, which is clearly observed in the tests at 55 ºC for 10 min, and at 75 ºC for one minute. While heat treatment at 55 ºC/10 min. resulted in a reduction of 5.3% (0.74 log CFU) in cell viability, a 15% loss of viability (1.41 log CFU, three times higher) was found in the tests with the free cells. Similarly, heating to 75 ºC for 1 min, demonstrated that encapsulation of the probiotic cells provided 2.3 times greater protection, with a 17.95% reduction in cell viability, in contrast to a reduction of 40.6% exhibited by the free cells. Even under the most drastic condition of heat treatments (75 ºC/10 min.), encapsulation contributed to maintaining 32.6% of viable cells, while free cells showed only 11.9% viability. It is interesting to note that by this heat treatment condition, a count in the range of 10^4^ log CFU of encapsulated *L. acidophilus* LA02 ID 1688 cells was found.

### Morphological aspects and FT-IR spectroscopy of the encapsulated probiotic cells

**Figure 1:**
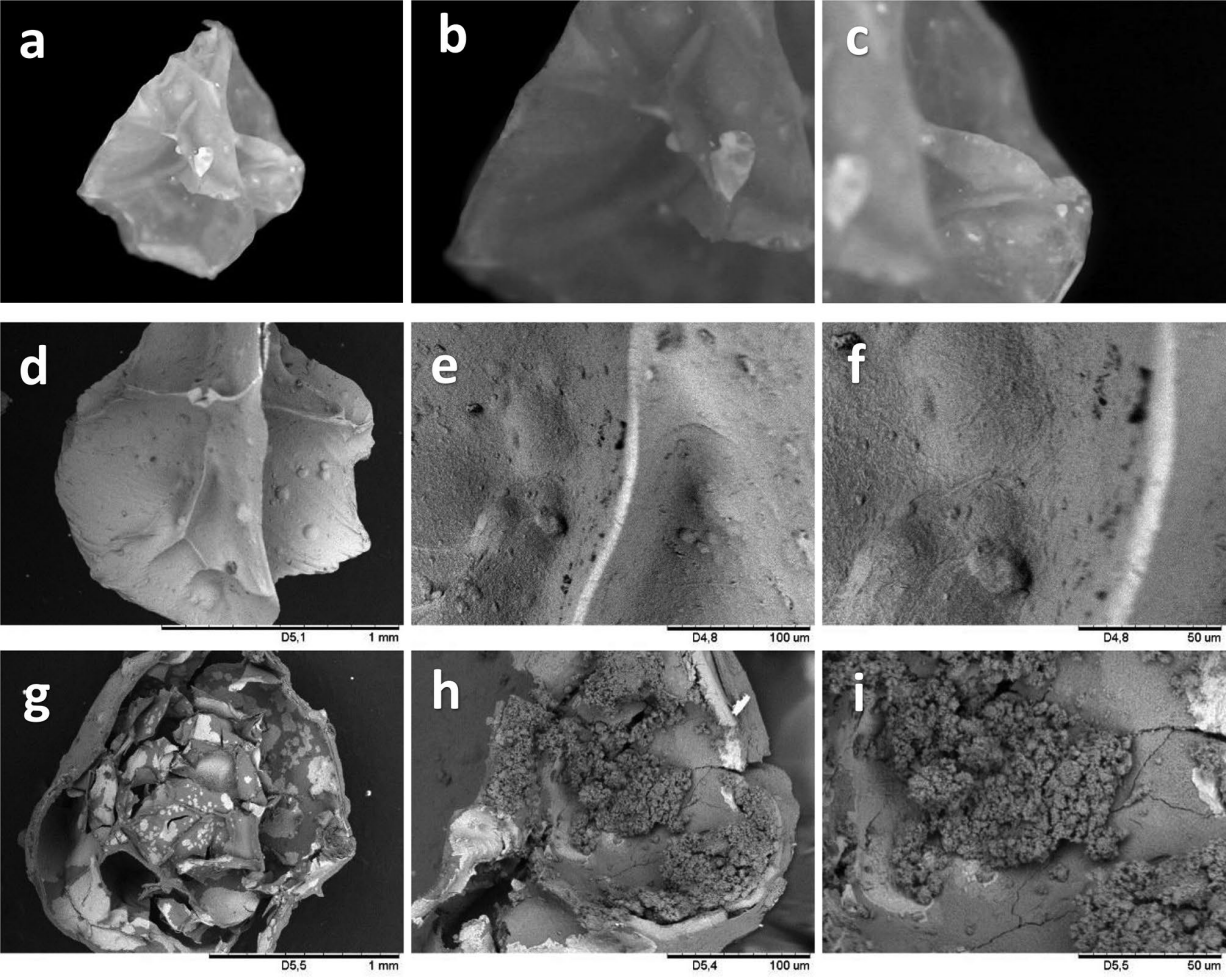
Micrographs of optical (**a–c**) and scanning electron (**d–i**) microscopy of the lyophilized calcium alginate capsules containing cells of the probiotic *Lactobacillus acidophilus* LA02 ID 1688. Magnification of ×50 (**a**); ×100 (**b,c**); ×100 (**d,g**); ×600 (**e,h**) and ×1200 (**f,i**).

The lyophilized calcium alginate capsules containing the entrapped probiotic cells presented an average diameter of 1.73 ± 0.16 mm (*p* value > 0.05, *p* = 0.141), with no significant statistical variation between the particle’s dimensions, which indicates that the encapsulation technique employed promoted obtaining spheres of a regular size. The images of optical microscopy and scanning electron microscopy (Fig. 1) reveal a compact structure, free from apparent cracks or breaks and a rough surface with grooves. The roughness and grooves formed were due to the loss of water by sublimation in the process of lyophilization of the spheres, which promoted a deformation of the spherical structure obtained immediately after the process of crosslinking sodium alginate in the calcium chloride solution.

According to Bassani et al*.*^24^ the appearance of grooves in alginate spheres can be attributed to the drying process. Dolly et al*.*^25^ described that during the freeze-drying process of polysaccharide hydrogel spheres, ice crystals occur due to the low temperatures to which these spheres are subjected in their preparation for lyophilization. After sublimation of these crystals under reduced pressure, a dry and porous matrix similar to a sponge is formed. Fareez et al*.*^26^ related the irregularity of the surface on the spheres to a higher concentration of polymer in specific areas of the encapsulated spheres.

The absence of cracks or ruptures on the external surface of the encapsulated spheres can contribute to greater protection of the internalized probiotic cells against permeability of gases, liquids or other materials, such as bile salts and stomach acid, as these features are directly related to loss of cell viability during the passage through the gastrointestinal tract. Figure 1g shows that the capsules have a macroporous internal structure, which is essential for trapping a larger number of probiotic cells. The formation of larger pores and cavities may be related to the use of magnesium hydroxide in the process to obtain hydrogel particles. Arenales-Sierra et al*.*^13^ reported that the use of magnesium hydroxide led to the production of spheres with larger pores and a less rigid alginate network, indicating that the presence of magnesium hydroxide affects the crosslinking of sodium alginate by calcium chloride.

The probiotic cells were entrapped inside the pores of the freeze-dried capsules as best seen at the 600× (Fig. 1h) and 1200× (Fig. 1i) magnifications. Apparently not all the pores of the macroporous structure of the calcium alginate matrix were filled with the probiotic cells, as can be seen in Fig. 1g. This aspect is possibly associated with the encapsulation efficiency of the hydrogel (89.4%) which, although high, many cells detached from the capsules during the encapsulation process, and therefore some pores were not filled with the probiotic cells.

It is noteworthy that a possible strategy for a better use of the macroporous structure of the matrix would be to cultivate the capsules containing the probiotic cells in MRS broth for cell proliferation within the pores before lyophilization of the capsules. This procedure could possibly allow obtaining capsules with a higher cell density.

**Figure 2:**
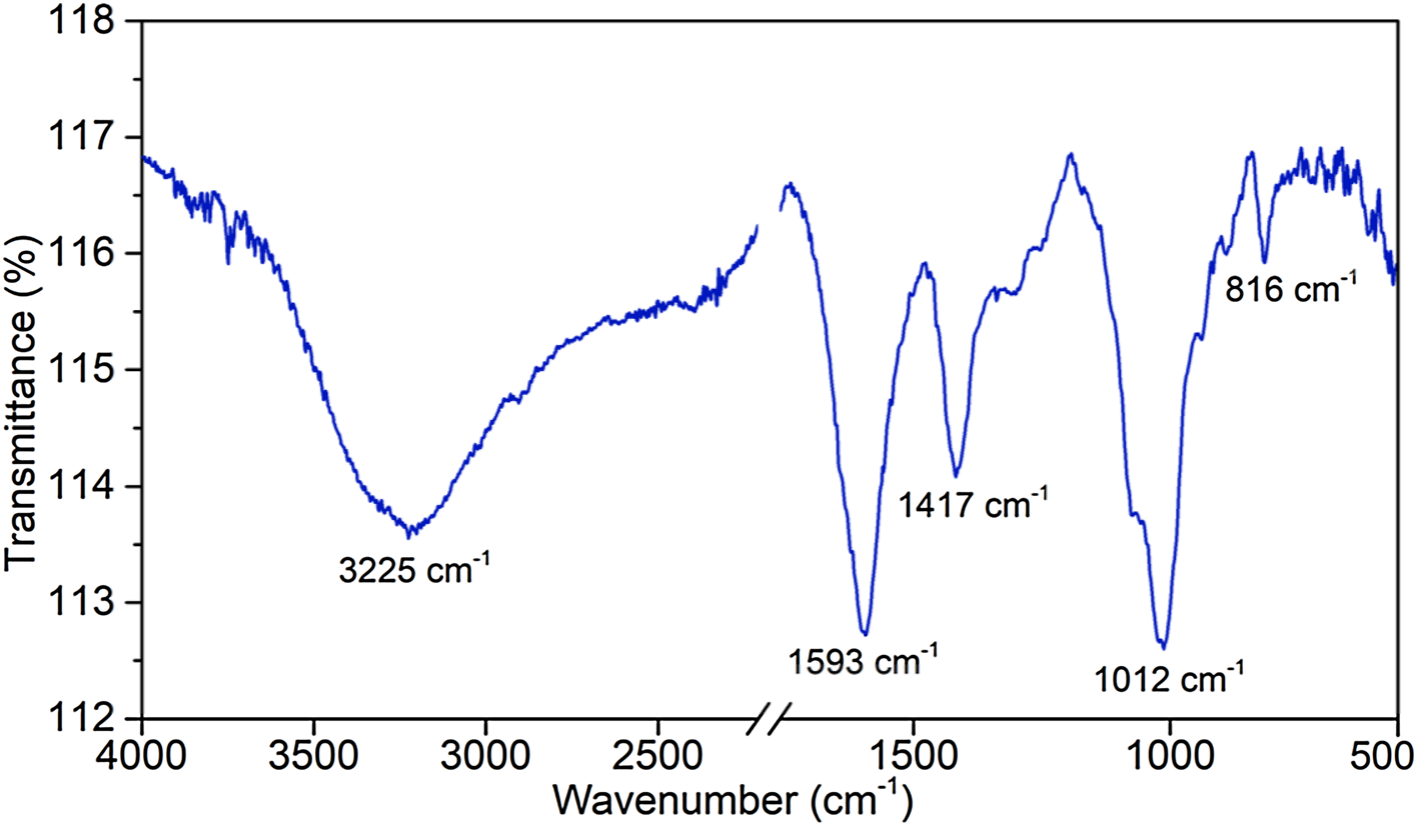
Fourier transform-infrared spectra of lyophilized hydrogel spheres containing *Lactobacillus acidophilus* la02 ID 1688 cells.

The FT-IR spectra of hydrogel capsules with the entrapped probiotic cells (Fig. 2) showed typical polysaccharide vibrational bands with alginate characteristic bands in the regions of 3225 cm^−1^, 1593 cm^−1^, 1417 cm^−1^, 1012 cm^−1^ and 816 cm^−1^. The band in the region of 3225 cm^−1^ is attributed to the stretching vibration of the hydroxyl groups (–OH) and the strong intensity of this band is due to the presence of many hydroxyl groups in the alginate structure. The weak intensity band in the region of 2905 cm^−1^ is attributed to the asymmetric stretching vibration of the CH_3_ and CH_2_ groups^16^. The band at 1593 cm^−1^ is related to the asymmetric stretching vibration of the connection between C–O of the COO– alginate group^27^. The intense absorption at 1593 cm^−1^ is also related to amide band I (stretching vibration C=O) of the functional groups of endogenous proteins ^16^, and vibration of amide II (C–N–H angular deformation in the plane and C–N stretch of probiotic cell proteins)^13^. The band at 1417 cm^−1^ is related to the symmetrical stretching vibration of the carboxyl group (C(=O)OH) (Nissola et al.^28^). The band at 1012 cm^−1^ is related to the symmetrical and asymmetric stretching of the C–O and C–O–C groups^13,16,29^. The absorptions in the regions between 1200 and 900 cm^−1^ can be attributed to the symmetric phosphorus-oxygen (P-O) stretching vibrations of the phosphodioxy group (PO_2_^−^) found in nucleic acids, and the vibration of C–O–C deformation of the polysaccharides belonging to cell membrane glycoproteins and lipopolysaccharides of the probiotic cells^16,30^. The band at 816 cm^−1^ is in a region (900 to 700 cm^−1^) called the true *fingerprint* region and contains very specific and weak spectral patterns of aromatic ring vibrations of aromatic amino acids (tyrosine, tryptophan, phenylalanine) and nucleotides^30^. Absorptions in this region are related to the presence of cellular material present in the encapsulated probiotic cells.

### Proximal and nutritional composition of *Pereskia aculeata* leaf flour and the formulated cereal bar

High contents of proteins (21.4%), dietary fiber (39%) and minerals (16.1%) were present in the flour of *P. aculeata* leaf (Table 2). *P. aculeata* leaves have been mentioned in the scientific literature as an interesting nutritional source, as they contain protein amounts (26% w/w) far higher than other vegetables commonly used as foods, such as beans, corn and cabbage^31^. The proteins of *P. aculeata* leaf flour are considered of good quality, presenting 85% digestibility^32,33^. However, according to Zem et al*.*^33^, *P. aculeata* flour provided as a single source of protein is inadequate for growth, although it is relevant for the maintenance of protein metabolism. According to these authors, it is a source of good quality protein due to the presence of few limiting essential amino acids, and meets the requirements of a diet for humans as recommended by FAO/WHO. Considerable levels of essential amino acids were present in the flour studied, viz., leucine (65.42 mg g^−1^), valine (48.83 mg g^−1^), lysine (48.13 mg g^−1^) threonine (44.86 mg g^−1^) isoleucine (37.15 mg g^−1^), tryptophan (18.93 mg g^−1^) and histidine (18.69 mg g^−1^) (Table 2).

**Table 2:** Proximal and nutritional composition of *Pereskia aculeata* leaf flour.

Proximal composition^a^ (*Pereskia aculeata* leaf flour)					
Moisture at 105 ºC	9.76 ± 0.08	Mineral residue	16.10 ± 0.05		
Total protein	21.40 ± 0.08	Dietary fiber	39.00 ± 0.87		
Total fats	1.65 ± 0.23	Carbohydrate	51.09 ± 0.03		
Calorific value		304.81 kcal/100 g			

Amino acid profile (*Pereskia aculeata* leaf flour)					
Essential^b^		FAO/WHO	Non-essential^b^		
			Aspartic acid	75.00 ± 0.33	
Phenylalanine	43.22 ± 1.65	–	Glutamic acid	100.0 ± 0.33	
Histidine	18.69 ± 0.66	93.5	Alanine	51.87 ± 2.64	
Isoleucine	37.15 ± 2.31	116.1	Arginine	46.73 ± 1.32	
Leucine	65.42 ± 1.98	99,1	Cystine	6.31 ± 0.33	
Lysine	48.13 ± 3.30	84.4	Glycine	48.13 ± 1.98	
Methionine	7.94 ± 0.66	–	OH-proline	4.44 ± 0.33	
Threonine	44.86 ± 1.32	144.7	Proline	39.95 ± 0.99	
Tryptophan	18.93 ± 0.99	222.6	Serina	38.32 ± 1.32	
Valine	48.83 ± 2.97	113.6	Aspartic acid	31.54 ± 0.33	

Sulfur amino acids^b^		FAO/WHO	Aromatic amino acids^b^		FAO/WHO
Methionine + cystine	14.2 ± 0.8	52.6	Phenylalanine + tyrosine	74.9 ± 1.6	144.0
**Monounsaturated fatty acids (MFA)**^**c**^					
Palmitoleic acid C16:1n7 (ω-7)	20.0 ± 1.0		Oleic acid C18:1n9c (ω-9)	160.0 ± 5.0	
**Polyunsaturated fatty acids (PFA)**^**c**^					
Linoleic acid C18:2n6c (ω-6)	270.0 ± 4.0		α-Linolenic acid C18:3n3 (ω-3)	640.0 ± 5.0	
**Saturated fatty acids (SFA)**^**c**^					
Lauric acid (C12:0)	10.0 ± 1.0		Stearic acid (C18:0)	85.0 ± 1.0	
Myristic acid (C14:0)	15.0 ± 1.0		Arachidonic acid (C20:0)	10.0 ± 0.0	
Palmitic acid (C16:0)	415.0 ± 6.0		Lignoceric acid (C24:0)	15.0 ± 1.0	
Margaric acid (C17:0)	10.0 ± 0.0		–	–	
**Total fats**^a^					
Monounsaturated	0.18 ± 0.06		Unsaturated	1.09 ± 0.15	
Polyunsaturated	0.91 ± 0.11		Saturated	0.56 ± 0.08	

^a^g per 100 g of *P. aculeata* flour (g 100 g^−1^).

^b^Milligrams per g of protein (mg g^−1^); chemical score in relation to the daily recommendation for adults (> 18 years).

^c^Milligrams per 100 g of flour (mg 100 g^−1^); recommendations by FAO: Food and Agriculture Organization, and WHO: World Health Organization.

The amino acid composition and the chemical score (CS) were compared for essential amino acids recommended by FAO/WHO for children aged between 6 months to 3 years^34^. The findings indicated that the sulfur amino acids (methionine and cystine, CS: 52.6) were the limiting amino acids. By contrast, tryptophan (CS: 222.6) was the essential amino acid with the highest CS and leucine (65.42 mg g^−1^), an essential amino acid was present in higher amounts.

Leucine and tryptophan are amino acids that can influence weight reduction, since they can act on controlling appetite^35–37^. Another positive aspect that should be mentioned is the low-fat content (1.65 g 100 g^−1^), with 66.1% of the total fat content corresponding to unsaturated fatty acids (Table 2).

*P. aculeata* flour was used as an ingredient in the cereal bar formulation with the main purpose of nutritional enrichment of the product, due to its high content of good quality protein and minerals. Most of the cereal bars available on the market are products designed to provide energy due to their carbohydrate content, and satiety due to their high fiber content. However, in recent years, the market has demanded more nutritionally complete products and, in this sense, the use of ingredients rich in proteins has been a good option.

Thus, *P. aculeata* flour was used as a proposal for a new low-cost protein ingredient in a high value-added product. There are still few reports in the literature about the industrial use of this unconventional edible plant that has great potential for use.

The formulated cereal bar showed high levels of carbohydrates (71.81 g 100 g^−1^), dietary fiber (19 g 100 g^−1^) and lipids (12.63 g 100 g^−1^), as well as a relevant protein content (5.44 g 100 g^−1^) (Table 3).

**Table 3:** Proximal and nutritional composition of the formulated cereal bar.

Proximal composition (cereal bar)				
	(g 100 g^−1^)	(g 20 g^−1^)^a^	DRV^b^ (%)	DRV^c^ (%)
Moisture at 105 °C	7.83 ± 0.08	1.57 ± 0.02	–	–
Total protein	5.44 ± 0.04	1.09 ± 0.01	10.88 ± 0.08	2.18 ± 0.02
Total fat	12.63 ± 0.16	2.53 ± 0.03	19.43 ± 0.25	3.89 ± 0.05
Mineral residue	2.29 ± 0.02	0.46 ± 0.01	–	–
Dietary fiber	19.00 ± 0.20	3.80 ± 0.04	76.00 ± 0.80	15.20 ± 0.16
Carbohydrates	71.81 ± 0.99	14.36 ± 0.20	23.94 ± 0.33	4.79 ± 0.07
Water activity	0.62 ± 0.01	0.62 ± 0.01	–	–
Calorific value (Kcal)	422.67 ± 5.56	84.53 ± 1.11	21.13 ± 0.28	4.23 ± 0.06

*DRV* daily reference value based on a diet of 2000 kcal in Brazil.

^a^Approximately weight of a cereal bar.

^b^Portion of 100 g.

^c^Portion of 20 g.

Carbohydrates were the components present in greater amounts in the formulated cereal bar, which arises from the addition of the agglutinating syrup used (cane molasses and brown sugar) (Table 3). Other sources of carbohydrates such as cereals (rice and oats) that contain β-glucans and hemicelluloses, and flour from *P. aculeata* and green banana (a source of resistant starch), as well as the chocolate coating also contributed to the total carbohydrate content in the formulation. In this context, it is worth mentioning cereal bars are products that are frequently consumed as sources of energy.

The fiber content present (19 g 100 g^−1^) characterizes the cereal bar as a product with an increased content of dietary fiber according to Brazilian legislation^38,39^. The 20-g portion of the product (i.e., one cereal bar) corresponds to 15.20% of the daily reference value (DRV) of dietary fiber intake based on a 2000 kcal diet.

The cereal bar showed a total fat content of 12.63 g 100 g^−1^, which corresponds to a DVR of 3.89% per 20 g portion based upon a 2000 kcal diet. The fats present in the bar comes from the chocolate coating (72% cocoa), as well as from the nuts used as an ingredient in the formulation. In addition to the nutritional quality, the probiotic potential of the product developed should be highlighted. The viability of the probiotic cells incorporated into the cereal bars was monitored over a period of 120 days storage at 25 ºC, and the results are presented in the Table 1 (see Supplementary Table S1 online).

The association of the probiotic encapsulation technique and the covering of the food bars with dark chocolate (72% cocoa) contributed to the maintenance of the viability of the probiotic cells during the storage period. In this context, Hossain et al*.*^40^ pointed out that the association of a chocolate coating with microencapsulated probiotic strains can be an excellent solution to protect the probiotic cells from environmental stresses, as our work also demonstrated.

A high percentage of cell survival was observed throughout the storage period (see Supplementary Table S1 online). Shortly after the incorporation of probiotic into the chocolate topping, a small reduction in cell viability (2%) was observed, which is possibly associated with possible physiological stress, which may be related to the temperature of the chocolate coating syrup (45 ºC to 50 ºC). However, throughout the storage period, the probiotic cells maintained high viabilities. After 60 and 90 days of storage, cell viability was maintained at levels close to 95% (10^13^ CFU) of the initial content of the probiotic cells incorporated into the product. At the end of the 4^th^ month (120 days), 90% of the probiotic cells added to the cereal bar was observed to remain viable, conferring a high probiotic potential on this food product (10^12^ CFU).

### Instrumental texture profile of the cereal bar

In the shear test an average force of 61.43 N was recorded, which represents the stress/force exerted when cutting the sample. As shown in Table 4, during the application of the shear force there were variations of intensity between 53.28 N and 77.25 N. Such variations are possibly associated with the composition of the product, which contains ingredients with different textures and, therefore, possess different resistances to cutting.

**Table 4:** Instrumental texture profile of the cereal bar.

Texture parameters	Observed values
Shear (N)	61.43 ± 8.06
Hardness (N)	161.02 ± 22.51
Adhesiveness (N mm)	1.25 ± 0.65
Elasticity (mm)	39.84 ± 19.52
Gumminess (N)	8.11 ± 2.49
Cohesiveness	0.05 ± 0.02
Chewiness (N)	3.94 ± 2.79

The instrumental test of hardness in food expresses the maximum compression force applied until the sample is deformed and/or ruptured. The maximum deformation force verified in the studied cereal bar was 161.02 N. Similar values of hardness were reported by Munhoz et al*.*^41^ in a cereal bar containing fruit pulp and bocaiuva almonds. These authors mentioned that products prepared with a high fiber content tend to result in denser and harder products, which we did not observe in the present work.

Adhesiveness represents the energy needed for food to separate from other materials. The cereal bar produced presented adhesion values that ranged from −1.80 to −0.77 N mm, with an average value of − 1.25 N mm. The low adhesion values may be associated with the chocolate that coated the product, as well as the presence of flours from green banana and *P. aculeata* that absorbed part of the agglutination syrup.

The elasticity parameter corresponds to the percentage of product recovery when deformed, i.e., the ability of a product to return to its original state after compression by the teeth^42^. The food bar had an average elasticity of 39.84 mm, with variations between 12.90 and 70.73 mm, which can be justified by the diversity of ingredients present in the formulation. The cohesiveness of a material represents the extent to which a material can be deformed before it breaks. This is related to the degree of compressibility that a material resists to the breaking point^42,43^. The cohesiveness value of the studied food matrix remained in the range of 0.03 to 0.08, indicating that the cereal bar did not present a high deformation capacity before rupture. This behavior may be associated with the presence of the flours (*P. aculeate* and green banana), which absorbed part of the agglutination syrup, making the food matrix more compact. The high fiber content also possibly contributed to a lower cohesiveness of the sample.

Chewability and gumminess correspond to the energy needed to perform the process of mastication and disintegrating a solid or semi-solid food, respectively, reducing its consistency to levels suitable for ingestion^43^. The chewiness and gumminess values were 3.94 N and 8.11 N, respectively. Values were lower than those reported by Muniz et al.^44^.

## Conclusion

The encapsulation technique developed using 2% (w/v) sodium alginate with added 0.1% (w/v) magnesium hydroxide as an encapsulating material, proved to be suitable for immobilizing *Lactobacillus acidophilus* LA02 ID 1688 cells, allowing the formation of capsules with high cell immobilization efficiency. The encapsulation technique was demonstrated to be promising for the protection of probiotic cells against gastric and intestinal environmental, contributing to the maintenance of high levels of cell viability. In addition, cell encapsulation associated with dispersion of the capsules in the chocolate coating was effective in increasing cell viability throughout the cereal bar’s shelf life.

The encapsulation protocol developed can be a good strategy for the introduction of probiotic cells in the diet, ensuring the delivery of viable cell quantities above that recommended by FAO/WHO, and guaranteeing the functional potential of the probiotic product.

## Supplementary Information

Supplementary Table S1.
