# Supplementary material for: Encapsulation and dispersion of Lactobacillus acidophilus in a chocolate coating as a strategy for maintaining cell viability in cereal bars
Source: Sci Rep. 2021 Oct 15;11:20550. doi: 10.1038/s41598-021-00077-0 (PMC8519969; doi:10.1038/s41598-021-00077-0)
Supplement: Supplementary file 1 — Supplementary Table S1. [file 41598_2021_77_MOESM1_ESM.docx]

**Table S1.** Viability of probiotic *Lactobacillus acidophilus* LA02 ID 1688 cells in the food bar during times of storage.

| **Time**  (days) | **Log CFU**^*^ | **Survival**^#^  (%) | **RLC**  (log CFU.g_­­_^-1^) |
| --- | --- | --- | --- |
| 0 | 13.63 ± 0.22^a^ | 98.27 | 0.24 |
| 30 | 13.51 ± 0.20^ab^ | 97.40 | 0.36 |
| 60 | 13.32 ± 0.13^b^ | 96.03 | 0.55 |
| 90 | 13.14 ± 0.12^bc^ | 94.74 | 0.73 |
| 120 | 12.54 ± 0.14^d^ | 90.41 | 1.33 |

^*^ Log of colony forming units per g of food bar; ^#^ Percentage survival of encapsulated probiotic cells in relation to the amount of cells added to the food bar; RLC: reduction of logarithmic cycles of the encapsulated cells over the storage time compared to the initial amount of cells. Means followed by distinct letters in the same column differ statistically (*p* <0.05).
